# Mu and Delta Opioid Receptors Oppositely Regulate Motor Impulsivity in the Signaled Nose Poke Task

**DOI:** 10.1371/journal.pone.0004410

**Published:** 2009-02-09

**Authors:** Mary C. Olmstead, Abdel-Mouttalib Ouagazzal, Brigitte L. Kieffer

**Affiliations:** 1 Department of Psychology, Queen's University, Kingston, Ontario, Canada; 2 Institut de Génétique et de Biologie Moléculaire et Cellulaire, Département Neurobiologie, Illkirch, France; Chiba University Center for Forensic Mental Health, Japan

## Abstract

Impulsivity is a primary feature of many psychiatric disorders, most notably attention deficit hyperactivity disorder and drug addiction. Impulsivity includes a number of processes such as the inability to delay gratification, the inability to withhold a motor response, or acting before all of the relevant information is available. These processes are mediated by neural systems that include dopamine, serotonin, norepinephrine, glutamate and cannabinoids. We examine, for the first time, the role of opioid systems in impulsivity by testing whether inactivation of the mu- (*Oprm1*) or delta- (*Oprd1*) opioid receptor gene alters motor impulsivity in mice. Wild-type and knockout mice were examined on either a pure C57BL6/J (BL6) or a hybrid 50% C57Bl/6J–50% 129Sv/pas (HYB) background. Mice were trained to respond for sucrose in a signaled nose poke task that provides independent measures of associative learning (responses to the reward-paired cue) and motor impulsivity (premature responses). *Oprm1* knockout mice displayed a remarkable decrease in motor impulsivity. This was observed on the two genetic backgrounds and did not result from impaired associative learning, as responses to the cue signaling reward did not differ across genotypes. Furthermore, mutant mice were insensitive to the effects of ethanol, which increased disinhibition and decreased conditioned responding in wild-type mice. In sharp contrast, mice lacking the *Oprd1* gene were more impulsive than controls. Again, mutant animals showed no deficit in associative learning. Ethanol completely disrupted performance in these animals. Together, our results suggest that mu-opioid receptors enhance, whereas delta-opioid receptors inhibit, motor impulsivity. This reveals an unanticipated contribution of endogenous opioid receptor activity to disinhibition. In a broader context, these data suggest that alterations in mu- or delta-opioid receptor function may contribute to impulse control disorders.

## Introduction

Impulsivity is a behavioral trait that varies across the general population. Extreme manifestations of impulsivity are revealed in a variety of pathological conditions including antisocial and borderline personality disorders, attention deficit hyperactivity disorder (ADHD), pathological gambling, eating disorders, obsessive-compulsive disorder and substance abuse [1]. Patients with neurological impairments such as Parkinson's disease, Schizophrenia, Tourette's syndrome and frontal lobe dementia also present with clinical features of impulsivity. The pervasiveness of this trait across patient populations, and the fact that it is a significant predictor of therapeutic efficacy for some disorders [2]–[4], provide compelling arguments for understanding the neuropharmacology of impulsivity.

Altered serotoninergic transmission has long been associated with impulse control disorders [5], and an important role for dopamine (DA) has been inferred from the therapeutic efficacy of psychostimulants in the treatment of ADHD [6]. Moreover, in humans, polymorphisms in serotonin (5-HT) and DA transporters or receptors is associated with ADHD and addiction [7]. Animal research has confirmed an important role for both DA and 5-HT in impulsivity, and also implicated norepinephrine, glutamate, and cannabinoid systems [8]. To date, no one has investigated the role of opioid systems in impulsivity using animal models.

Opioid receptors have been studied extensively in relationship to drug abuse. The opioid system consists of endogenous neuropeptides that produce effects by acting at mu, delta and kappa opioid receptors. Deletion of the mu-opioid receptor gene (*Oprm1* ^−/−^) in mice reduces or eliminates the rewarding properties of opioids as well as non-opioid drugs such as ethanol, cocaine, nicotine and cannabinoids. The consistency of behavioral effects across different classes of abused drugs led to the hypothesis that mu-opioid receptors represent a gateway to drug addiction [9]. Unlike mu-opioid receptor knockout mutants, mice lacking the delta opioid receptor gene (*Oprd1^−/−^*) show intact cannabinoid-induced reward [10] and increased self-administration of ethanol [11]. These mice exhibit increased depressive-like behaviors and higher basal anxiety levels [12], the latter of which is reversed following ethanol intake [11]. Delta opioid receptors, therefore, influence emotional responses and this, in turn, impacts on drug taking behaviors.

**Figure 1:**
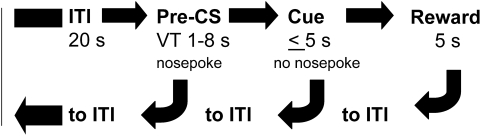
The progression of each trial during phase 4 of the signaled nose poke task. Each trial started with a 20 s ITI; responses during this period had no consequence. Nose pokes during the subsequent pre-CS period (VT 1–8 s) reinstituted the ITI. If mice refrained from responding during the pre-CS period the trial progressed to the 5 s cue presentation. Nose pokes in the pre-assigned reinforcement hole during the cue presentation turned off the cue and immediately elevated the sucrose dipper. Nose pokes in the non-reinforced hole had no consequence. CS = conditioned stimulus; ITI = inter-trial interval; VT = variable time.

Drug addiction is a complex state which results from gradual adaptations of the brain to repeated drug exposure. The rewarding properties of drugs promote initial drug use when drugs are consumed voluntarily but, as the disorder develops, addicts lose control of their behavior and drug intake becomes independent of drug reward [13], [14]. Impulsivity is a critical feature of this state in that addicts are unable to inhibit their drug-taking responses even if the subsequent rewarding effects of the drug are minimal. The question then arises, whether mu- and delta-opioid receptors, in addition to their roles in reward and emotional processing, also regulate impulsivity. Mu and delta knockout animals exhibit marked opposing phenotypes in a number of tasks (locomotor activity, dark-light box, elevated plus maze, forced swim); in contrast, kappa receptor knockout mice are comparable to wild-type controls in these tests [12]. Thus, as a first step in studying the role of opioid receptors to motor impulsivity, we elected to examine mu and delta mutant lines. To this aim, we tested whether deletion of the *Oprm1* or the *Oprd1* gene alters motor impulsivity in mice. We used a signaled nose poke task [15] that provides independent measures of impulsivity (the inability to withhold a prepotent response) and conditioned reward (approach responses to a light previously paired with a sucrose reward). Mice are required to withhold responding for a liquid sucrose reward until a visual cue is presented (Fig. 1). Impulsivity in this task is comparable to premature responding in the 5-choice serial reaction time task or disinhibition in the go/no-go task, two standard measures of impulsivity in rodents. Compared to these other tests, however, the signaled nose poke task is acquired rapidly and optimal performance does not rely as heavily on attentional (5-choice) or discriminative (go/no-go) abilities. Importantly, performance in the signaled nose poke task seldom reaches asymptotic levels, allowing both increases and decreases in impulsivity to be easily detected. Given the role of endogeneous opioids in the neuropharmacological effects of ethanol [16], and the common assumption that ethanol intoxication is associated with impulsive behavior in humans [17], we performed one additional test in which we evaluated whether ethanol alters performance of wild-type or knockout mice in the signaled nose poke task.

## Materials and Methods

### Subjects

Mu- and delta-opioid receptor knockout mice and their wild-type controls were bred in-house and genotyped a few days after birth. Mice were bred on either a hybrid 50% 129SVPas 50% C57Bl6J background (HYB), (the original background of opioid receptor knockout mice) or backcrossed onto the C57Bl6J background (classically used in mouse behavioral studies) for more than 12 generations (BL6). A detailed description of the construction of these lines and their genotyping has been described previously [12], [18].

At 112–126 days of age, mice were transferred from the breeding facility to the phenotyping centre where they were group housed on a 12-h light/dark cycle with lights on at 700 am. Food was available *ad libitum* throughout the experiment; with the exceptions noted below; access to water was restricted to 2 h per day beginning 2–4 h after testing. Behavioral testing commenced at 17–20 weeks of age and finished at 26–33 weeks of age. All animals were weighed twice per week; there were no significant group differences in weight across the experiment. Animal care was conducted in accordance with the European Communities Council Directive of 24 November 1986 (86/609/EEC) and the experiments were approved by the Comité régional d'éthique en matière d'experimentation animale de Strasbourg, CREMEAS, 2003-10-08-[1]-58.

### Apparatus

Impulsivity testing was conducted in operant boxes (26.5×22.0×20.0 cm) housed in a sound-attenuating chamber (Coulbourn Instruments, Allentown PA). Each box was fitted with two nose poke response holes, a cue light, a houselight and a liquid dipper that was accessed through a recessed magazine. Sucrose delivery was signaled by an infrared light located 4 cm above the magazine. Nose pokes and entries to the magazine were detected by infrared beams crossing each opening vertically. The control of stimuli and recording of responses were managed by an IBM-type computer using Graphic State Notation 2 software.

Sucrose and water consumption were measured in 24 automated chambers (11×22×19 cm) made of wire mesh floor and Plexiglas sidewalls (Imetronic, Pessac, France). Each cage was fitted with infra-red captors located 2 and 8.5 cm from the floor, allowing measurement of ambulatory locomotor activity and rears, respectively. Water and sucrose consumption were measured using an automated lickometer.

### Behavioral procedures

#### Signaled nose poke task

Mice were trained in the signaled nose poke task as described previously [15] with minor modifications. These included the cue signaling reward availability (5 s visual stimulus rather than a 3 s auditory stimulus) and the reward (5 s presentation of liquid sucrose rather than the delivery of a single sucrose pellet). The sessions were increased from 30 to 40 min to accommodate these changes.

One day prior to magazine training, and following 21 h of water deprivation, a sucrose solution (25% w/v) was presented in the home cage for one hour. Beginning the next day, all mice underwent magazine training (2 sessions) consisting of 30 presentations of liquid sucrose, presented at random intervals approximately every 30 seconds. During sucrose presentation, the houselight was turned off and the magazine light was turned on. Magazine entries were recorded and used as an indicator that mice had learned the location of the sucrose reward. The liquid dipper remained elevated for 5 s after a magazine entry to a maximum of 20 s per sucrose presentation.

Subsequent training was conducted in 4 phases with one training session per day. In Phase 1, mice were reinforced for nose poking on a fixed-ratio (FR1) schedule of reinforcement on either the right or left side (assignment of reinforced hole counterbalanced across groups). The discriminative stimulus (i.e., the visual cue) was turned on throughout these sessions, except during presentation of the reinforcer (5–20 s elevation of the sucrose dipper). Training continued until 25 reinforcers were presented in one session. In Phase 2, response requirements were increased to an FR3 schedule and the elevation of the sucrose dipper was limited to 5 s. Training continued until 25 reinforcers were presented in one session. In Phase 3, the discriminative stimulus was reduced to 5 s and presented 50 times with a fixed inter-trial-interval (ITI) of 30 s; only those responses occurring on the reinforced side during the cue were reinforced (FR1) by a 5 s elevation of the sucrose dipper. Training progressed to Phase 4 when animals made 10 reinforced nose pokes (i.e., during the cue) in one session. Animals that did not reach the training criterion in Phases 1, 2, or 3 after 20 sessions were removed from the experiment, and data from these sessions was not included in the statistical analyses. In Phase 4, the ITI was set at 20 s, followed by a pre-cue interval of 1–8 s. Phase 4 testing continued for 10 sessions. Nose pokes in the presence of the discriminative stimulus immediately turned off the visual cue and elevated the sucrose dipper (5 s). In one final session, the duration of the cue presentation was increased to a maximum of 30 s per trial. Thus, mice had up to 30 s to detect and respond to the visual cue before the next ITI was initiated.

#### Ethanol administration

Following the completion of Phase 4 testing, mice were retested in the signaled nose poke task following an injection of ethanol (0, 0.75, 1.25, 1.75 g/kg i.p.; 20% w/v in isotonic saline). Doses were administered in ascending or descending order (counterbalanced within groups) and all animals were tested twice at each dose. A minimum of 48 h separated each test.

#### Sucrose Preference

At the end of all behavioral tests, sucrose and water intake were assessed for 24 h, beginning at 11:00 during the light cycle (lights off at 19:00). Water and sucrose (25%) were provided in two bottles at the front of the cage and the number of licks was automatically scored for each bottle and measured as a function of time. Regular chow was freely available throughout the session. Sucrose preference was calculated for each phase as a percentage of total fluid intake (sucrose and water combined). Locomotor activity was measured as horizontal beam breaks at the front and back of the cage.

### Statistical analyses

The same wild-type mice on a BL6 background were used the control group for both mu and delta opioid receptor knockout mice; separate groups of HYB wild-type animals were used as controls of *Oprm1* ^−/−^ and *Oprd1* ^−/−^ groups on a HYB background. Data were then analyzed separately (*Oprm1* ^−/−^ and *Oprd1* ^−/−^) using an analysis of variance (ANOVA) with genotype (wild-type versus knockout) and background (BL6 versus HYB) as a between-subjects factors and session as a within-subjects factor (Magazine training in Phase 4, and Ethanol testing sessions). Post-hoc analyses were conducted using Tukey's test. Whenever there were violations of sphericity, P-values from the Greenhouse-Geisser correction to the within-group degrees of freedom were reported for all ANOVA tests. During Magazine training, the total number of magazine entries, magazine entries during sucrose presentation and the % of total magazine entries that occurred during sucrose presentations were analyzed. The dependent variable in Phases 1–3 was days to criterion. In Phase 4 and during Ethanol testing, the conditioned responses (CR; reinforcers/cue presentations) and efficiency ratio (reinforcers/nose pokes) were dependent variables. Appetitive learning is reflected in the CR measure whereas impulsivity is reflected in the efficiency ratio. Data from the ethanol sessions were analyzed with genotype and background as between-subjects factors and dose as a repeated factor. There was no effect of repeated testing on efficiency ratios or CR so data were collapsed across this factor. In all sessions, the number of responses on the non-reinforced side, the number of magazine entries during reward and non-reward periods, the latency to enter the magazine following elevation of the sucrose dipper, were recorded. Sucrose preference data were analyzed using a two-way ANOVA (genotype x background).

## Results

### Training

Mice from the six groups progressed through the training phases of the signaled nose poke task at the same rate (Table 1). All animals learned the significance of the reward presentation, evidenced by an increase in the proportion of magazine entries that occurred during sucrose availability: there were no genotype or background differences on this measure. Nor were there any genotype, background or interaction effects on the days to criterion measure in Phases 1–3 (all Fs<1.2; NS), indicating that deletion of neither mu-opioid nor delta-opioid receptors affected learning on this task.

**Table 1:** Training on the signaled nose poke task.

Group	Magazine Training		Phase 1: FR1	Phase 2: FR3	Phase 3: ITI
	Proportion Reward		Days to Criterion		
wild-type HYB	.38±.06	.54±.04	3.5±.35	3.20±.31	5.70±.41
*Oprm1 ^−/−^* HYB	.44±.05	.51±.04	4.0±.49	2.70±.47	5.20±.67
*Oprd1 ^−/−^* HYB	.42±.10	.46±.04	2.88±.64	3.87±.64	6.12±.58
wild-type BL6	.41±.07	.59±.06	4.0±.57	3.17±.70	6.25±.52
*Oprm1 ^−/−^* BL6	.48±.03	.54±.05	4.4±.60	3.80±.39	5.50±.62
*Oprd1 ^−/−^* BL6	.53±.03	.59±.09	3.33±.41	4.00±.67	6.00±.73

The first two columns display the proportion (±SEM) of magazine entries that occurred during elevation of the sucrose dipper across two sessions of magazine training. The last three columns display the days to criterion measure for Phases 1–3 during task training. With fixed ratio (FR) responding in Phases 1 and 2, the criterion to progress to the next phase was 25 reinforcers earned per 40-min session. With the introduction of the inter-trial interval (ITI) in Phase 3, this was reduced to 10 reinforcers per session. There were no significance differences between knockout and wild-type animals on any measure.

wild-type HYB, *n* = 20; *Oprm1* ^−/−^ HYB, *n* = 10; *Oprd1 ^−/−^* HYB, *n = *8*;*

wild-type BL6, *n* = 24; *Oprm1* ^−/−^ BL6, *n* = 11; *Oprd1 ^−/−^* BL6, *n = *10

### Testing

#### Deletion of the mu opioid receptor gene decreases motor impulsivity

**Figure 2:**
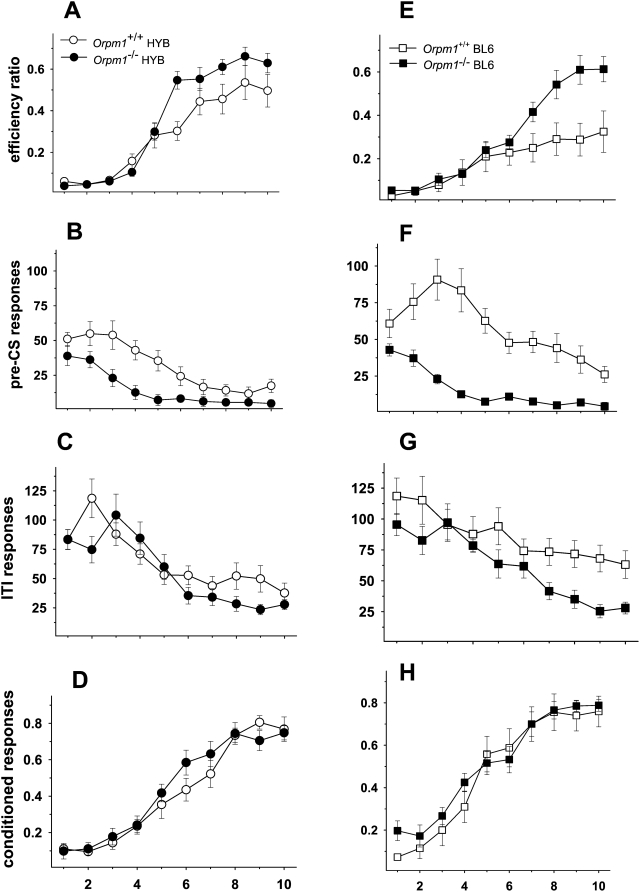
*Oprm1* ^−/−^ mice perform better in the signaled nose poke task. Performance on Phase 4 of the signaled nose poke task for mu-opioid receptor knockout mice and their wild-type controls on a HYB (A–D) and BL6 (E–H) backgrounds. A and E: Efficiency ratios (rewards/nose pokes) increased across sessions with *Oprm1 ^−/−^* mice on both genetic backgrounds performing significantly better than wild-type controls. Mice lacking the *Oprm1* gene exhibited lower levels of responding throughout Phase 4 testing; this phenotypic difference was most apparent during the pre-CS period (Fig. 2B and 2F), when animals must learn to inhibit responding in order to maximize the number of rewards earned. C and G: Responses on the rewarded side during the 20-s inter-trial interval (ITI) decreased across sessions with lower overall responses in the *Oprm1^−/−^* groups and in the HYB strain. D and H: Conditioned responding (rewards/signals) increased across sessions with no genotype or background differences. *Oprm1* ^+/+^ HYB, *n* = 11; *Oprm1* ^+/+^ BL6, *n* = 12; *Oprm1* ^−/−^ HYB, *n* = 10; *Oprm1* ^−/−^ BL6, *n* = 11.

Fig. 2 shows the effect of mu-opioid receptor deletion on testing in the signaled nose poke task. In contrast to the training data, *Oprm1 ^−/−^* mice on both genetic backgrounds performed significantly better than wild-type controls during Phase 4 testing. Efficiency ratios (rewards/nose pokes) increased across 10 sessions [F(9,351) = 59.82, p<.01] verifying that all groups were capable of learning the task (Fig. 2A and 2E). The significant 3-way interaction [F(9,351) = 2.05, p<.05] indicated that the difference in efficiency ratios between wild-type and knockout mice depended on the session and the background strain. Pairwise comparisons revealed that HYB, but not BL6, knockout animals differed from their wild-type controls on session 6. In sessions 7–10, BL6 knockout mice performed better than controls, whereas HYB knockout mice showed higher efficiency ratios only on session 8. The significant genotype [F(1,39) = 8.79, p<.01] and background [F(1,39) = 6.73, p = .05] differences were due to higher efficiency ratios in both *Oprm1 ^−/−^* groups compared to their own wild-type control, as well as higher efficiency ratios in the HYB background.

As seen in Fig. 2B and 2F, responses on the rewarded side during the pre-CS period (variable time 1–8 s) showed a general decline across sessions, [F(9,351) = 19.49, p<.01] and an interaction of this measure with genotype [F(9,351) = 5.75, p<.01]. There were significant genotype [F(1,39) = 15.3, p<.01] and background [F(1,39) = 6.98, p<.01] differences due to reduced pre-CS responding in the *Oprm1^−/−^* groups and in the HYB strain.

Responses on the rewarded side during the 20-s inter-trial interval (ITI) showed a similar pattern [F(9,351) = 17.11, p<.01], but the rate of decrease differed across groups [F(9,351) = 2.77, p<.05] (Fig. 2C and 2G). Responding was similar in all four groups during the initial sessions, but declined to a lower level in the *Oprm1 ^−/−^* groups by the end of the sessions. The significant main effects of genotype [F(1,39) = 8.2, p<.01] and background [F(1,39) = 4.81, p<.05] reflected lower overall responses in the *Oprm1^−/−^* groups and in the HYB strain.

Finally, conditioned responding (rewards/signals) increased across sessions [F(9,351) = 119.51, p<.01], but there were no genotype or background differences on this measure and no statistically significant interactions (Fig. 2D and 2H).

#### Deletion of the delta opioid receptor gene increases motor impulsivity

**Figure 3:**
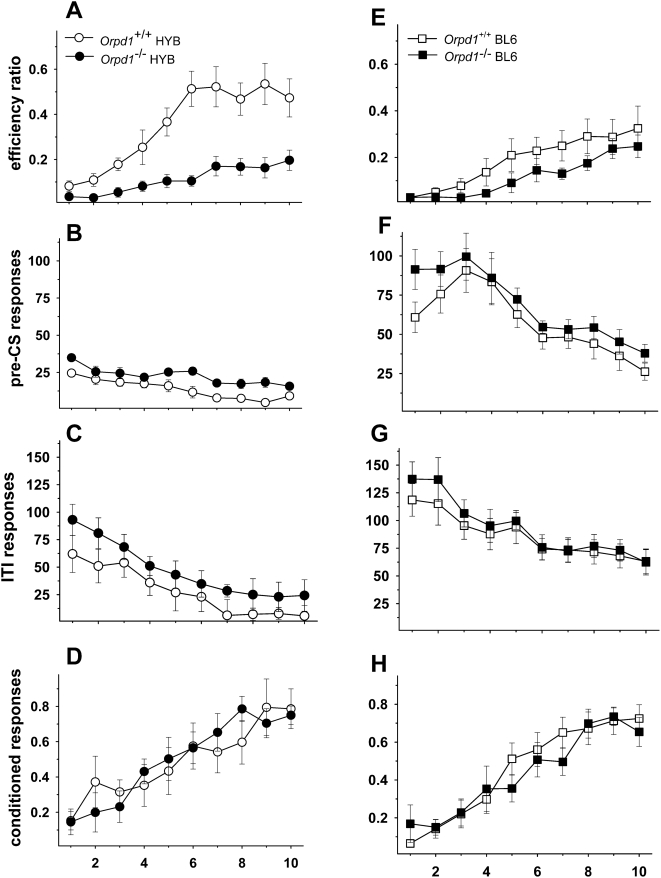
*Oprd1* ^−/−^ mice are impaired in the signaled nose poke task. Performance on Phase 4 of the signaled nose poke task for delta-opioid receptor knockout mice and their wild-type controls on a HYB (A–D) or BL6 (E–H) background. A: Efficiency ratios (rewards/nosepokes) increased across 10 sessions indicating that all groups were capable of learning the task. Both HYB and BL6 *Oprd1 ^−/−^* mice exhibited lower efficiency ratios (i.e., more impulsivity) than their wild-type controls. The significant background effect reflected the fact that both knockout and wild-type BL6 mice were more impulsive than mice on a HYB background. B and F: Responses during the pre-CS period also declined across sessions although the effect was not as pronounced in *Oprd1 ^−/−^* mice. Again, responses of HYB mice were increased compared to mice on a BL6 background. C and G: Responses during the 20-s inter-trial interval (ITI) decreased across sessions and the rate of decline was consistent across groups. Nonetheless, HYB mice responded at higher rates than BL6 mice of both genotypes throughout the sessions. D and H: Conditioned responding (rewards/signals) increased across sessions, but there were no genotype or background differences on this measure and no statistically significant interactions. *Oprd1^+/+^* HYB, *n = *9*; Oprd1^ +/+^* BL6, *n* = 12; *Oprd1 ^−/−^* HYB, *n = *8*; Oprd1 ^−/−^* BL6, *n = *10.

Fig. 3 shows the effect of delta opioid receptor deletion on testing in the signaled nose poke task. Similar to the mu-opioid receptor knockout animals, efficiency ratios increased across the 10 testing sessions of Phase 4 [F(9,315) = 26.76, p<.01], indicating that all groups were capable of learning the task (Fig. 3A and 3E). The genotype x session interaction [F(9,315) = 3.78, p<.01] and the main effect of genotype [F(1,35) = 11.55, p<.01] were the result of both *Oprd1^−/−^* groups achieving lower efficiency ratios (i.e., increased impulsivity) compared to their respective control groups (post-hoc tests p<0.05). In addition, as in the *Oprm1^−/−^* experiment, there was a significant difference in the efficiency ratios of the two wild-type strains, with the HYB animals exhibiting significantly higher efficiency ratios in sessions 3–10. The session x background and session x background x genotype interactions were not statistically significant.

Both the genotype [F(1,35) = 4.32, p<.05] and background [F(1,35) = 87.34, p<.01] differences in responding were apparent during the 1–8 s pre-CS period when animals must learn to inhibit their responses (Fig. 3B and 3F). Pre-CS responses declined across sessions [F(9,315) = 14.4, p<.01] and the effect was more pronounced in BL6 mice of both genotypes [F(9,315) = 6.3, p<.01]. Responses also decreased during the ITI period [F(9,315) = 20.24, p<.01] but this factor did not interact with group or genotype (Fig. 3C and 3G). Unlike pre-CS responses, there were no group differences in responding during the ITI period [F(1,35) = 3.15, p>.05], although the HYB animals continued to respond at higher rates during this period [F(1,35) = 61.45, p<.01]. Thus, increased responding in the signaled nose poke task by *Oprd1 ^−/−^* mice was confined to the pre-CS period indicating that these mice are incapable of refraining from making an anticipated response.

The proportion of CRs also increased across sessions [F(9,315) = 123.29, p<.01] (Fig. 3D and 3H), but there were no significant genotype or background differences on this measure and none of the interactions were statistically significant. Thus, in contrast to the impulsivity measure, the ability to learn a conditioned responses was not disrupted by deletion of the *Oprd1* gene.

In sum, mice lacking mu-opioid receptors were significantly better at inhibiting a motor response, whereas mice lacking the delta-opioid receptor were significantly worse.

It is unlikely that these changes in efficiency ratios simply reflect a general reduction in responding because there were no genotypic differences in: 1) responses on the non-reinforced side throughout training and testing (Table 2, left columns) or 2) responses on the reinforced side during the final sessions of Phases 1–3 or the first session of Phase 4 (Table 2, right columns). In addition, conditioned responses to the reward-paired cue were similar across genotype and strain suggesting that differences in associative learning did not impact on task performance. To evaluate possible differences in attentional processes, we increased the duration of the visual cue from 5 to 30 s during a final test session. This manipulation did not affect efficiency ratios or conditioned responses in any group (data not shown). The mean latency to respond to the visual cue in this final session was 2.28 s and less than 2% of the cue presentations reached the maximum 30 s duration; there were no genotype or background differences on either measure. Thus, it is unlikely that the decreased impulsivity in *Oprm1 ^−/−^* mice or the increased impulsivity in *Oprd1 ^−/−^* mice reflects alterations in attentional processes. Finally, sucrose preference was similar in wild-type and knockout mice, and there were no strain differences in this measure, ruling out the possibility that differential responses to the reward influenced our findings (mean sucrose preference across groups = 94.7%).

**Table 2:** Nosepoke responses on the signaled nose task.

Group	Phase 1		Phase 2		Phase 3		Phase 4	
SNP	non-RF	Total	non-RF	Total	non-RF	total	non-RF	total
*Oprm1^ +/+^* HYB	19.09 (3.08)	67.27 (7.73)	11.55 (2.48)	144.45 (15.32)	5.72 (1.67)	181.36 (10.23)	3.20 (0.89)	143.10 (10.79)
*Oprm1 ^−/−^* HYB	21.75 (3.77)	76.40 (9.49)	13.16 (3.03)	136.10 (16.89)	9.25 (2.48)	198.20 (13.91)	5.49 (1.75)	127.80 (11.87)
*Oprm1^ +/+^* BL6	25.50 (5.80)	57.52 (8.04)	16.93 (3.65)	115.17 (12.5)	10.16 (2.72)	188.75 (19.99)	6.78 (2.12)	183.91 (20.44)
*Oprm1 ^−/−^* BL6	22.10 (4.29)	51.50 (8.49)	17.61 (4.74)	116.70 (15.42)	8.70 (2.50)	166.80 (15.63)	4.45 (1.77)	146.10 (11.61)
*Oprd1^ +/+^* HYB	16.77 (2.63)	56.33 (8.70)	12.88 (2.24)	100.67 (11.23)	7.44 (2.02)	150.44 (18.74)	4.00 (1.36)	151.33 (18.75)
*Oprd1 ^−/−^* HYB	20.75 (3.98)	69.00 (8.75)	15.00 (2.0)	87.13 (9.17)	13.00 (3.68)	155.25 (20.46)	6.25 (1.36)	149.50 (15.36)
*Oprd1^ +/+^* BL6	25.50 (5.80)	57.52 (8.04)	16.93 (3.65)	115.17 (12.5)	10.16 (2.72)	188.75 (19.99)	6.78 (2.12)	183.91 (20.44)
*Oprd1 ^−/−^* BL6	18.20 (3.78)	86.90 (9.69)	19.70 (3.33)	135.7 (15.36)	19.90 (3.81)	158.00 (13.81)	9.00 (2.17)	182.80 (10.51)

The two columns under each phase display mean nose pokes (SEM) in the non-reinforced (non-RF) hole (left column) and total nose pokes (right column) during the final session of Phases 1–3 (i.e., the day the animals met criterion) and the first session of Phase 4. Note that the number of sessions in Phases 1–3 varied for each animal, depending on the days to reach criterion. There were no significance differences between knockout and wild-type animals on any measure.

*Oprm1* ^+/+^ HYB, *n* = 11; *Oprm1* ^−/−^ HYB, *n* = 10; *Oprm1* ^+/+^ BL6, *n* = 12;

*Oprm1* ^−/−^ BL6, *n* = 11

Oprd1^+/+^ HYB, n = 9; Oprd1 ^−/−^ HYB, n = 8; Oprd1^ +/+^ BL6, n = 12;

Oprd1 ^−/−^ BL6, n = 10

#### Ethanol administration alters performance in delta but not mu opioid receptor knockout mice

**Figure 4:**
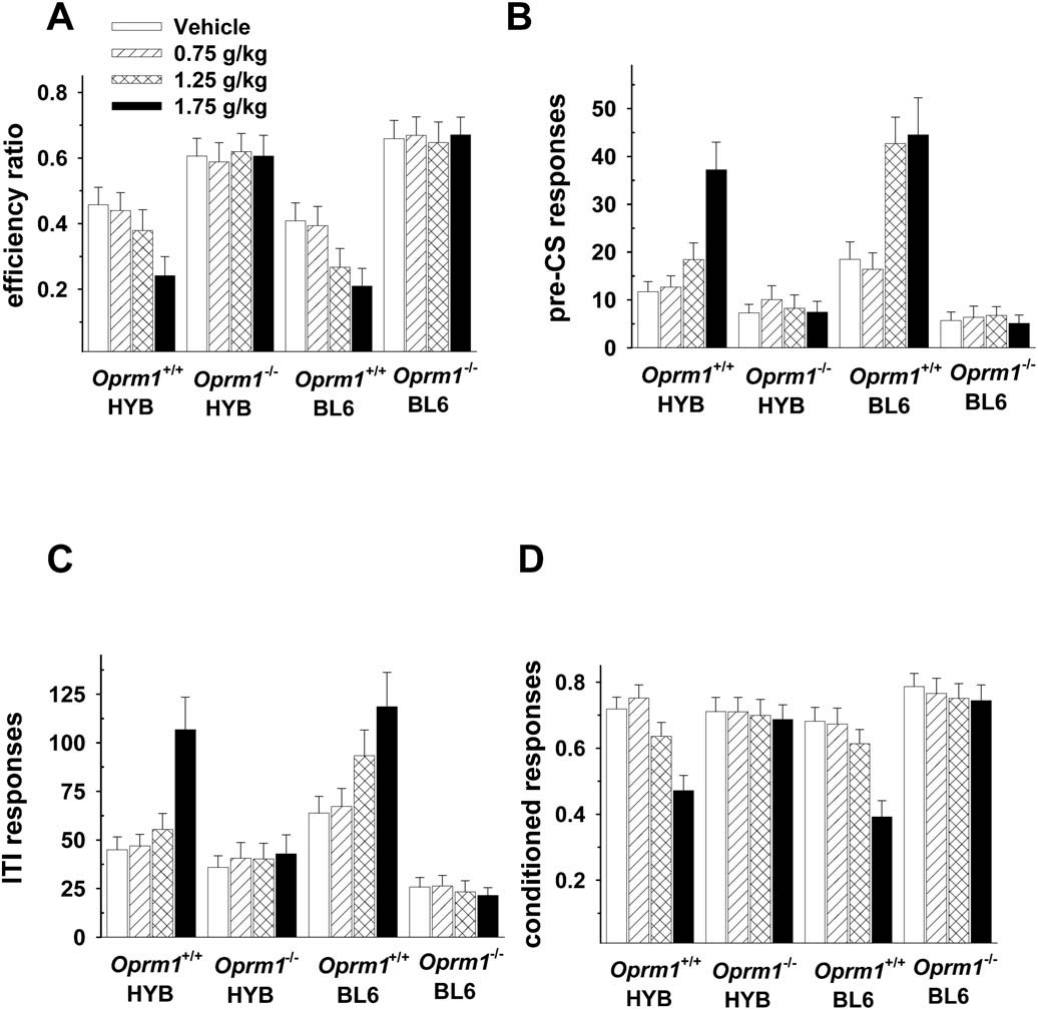
*Oprm1* ^−/−^ mice are not affected by ethanol in the signaled nose poke task. A: Efficiency ratios (rewards/nosepokes) decreased with increasing doses of ethanol, but only in wild-type mice. B: The same effect was observed during the pre-CS period. There were no significant strain differences on any behavioral measure during ethanol testing, nor were there any strain-dose interactions. C: Ethanol increased nose pokes that occurred during the inter-trial interval (ITI) in wild-type but not knockout animals. D: Ethanol also altered conditioned responses in *Oprm1* ^+/+^ , but not *Oprm1* ^−/−^ mice. The highest dose of ethanol (1.75 g/kg) produced the greatest effect on conditioned responses in wild-type mice. *Oprm1* ^+/+^ HYB, *n* = 11; *Oprm1* ^+/+^ BL6, *n* = 12; *Oprm1* ^−/−^ HYB, *n* = 10; *Oprm1* ^−/−^ BL6, *n* = 11.

Ethanol administration decreased efficiency ratios and conditioned responses in wild-type mice but had no effect on mice lacking mu-opioid receptors (Fig. 4).

As see in Fig. 4A, the effect of ethanol on efficiency ratios was dependent on dose [F(3,246) = 5.95, p<.01] and genotype [F(1,82) = 38.74, p<.01] as well as an interaction between the two [F(1,82) = 13.61, p<.01. Post-hoc tests indicated that these effects were due to higher efficiency ratios in *Oprm1* ^−/−^ animals and an ethanol-induced decrease in efficiency ratios only in *Oprm1^ +/+^* mice. An analysis of pre-CS responses (Fig. 4B) revealed a significant effect of dose [F(3,246) = 28.6, p<.01], genotype [F(1,82) = 48.3, p<.01] and a dose x genotype interaction [F(3,246) = 17.58, p<.01]. There were no significant strain differences on any behavioral measure during ethanol testing. A similar effect was observed during the ITI (Fig. 4C): ethanol altered responding [F(3,246) = 14.06, p<.01] and this effect interacted with genotype [F(3,246) = 13.98, p<.01]. The main effect of genotype [F(1,82) = 104.95, p<.01] confirmed that *Oprm1* ^−/−^ animals were responding at lower rates across doses. Ethanol also had differential effects on conditioned responses in *Oprm1* ^−/−^ and *Oprm1* ^+/+^ mice (Fig. 4D). This was confirmed by significant main effects of dose [F(3,246) = 37.58, p<.01] and genotype [F(1,82) = 8.95, p<.05] and an interaction between the two [F(1,82) = 33.85, p<.01].

**Figure 5:**
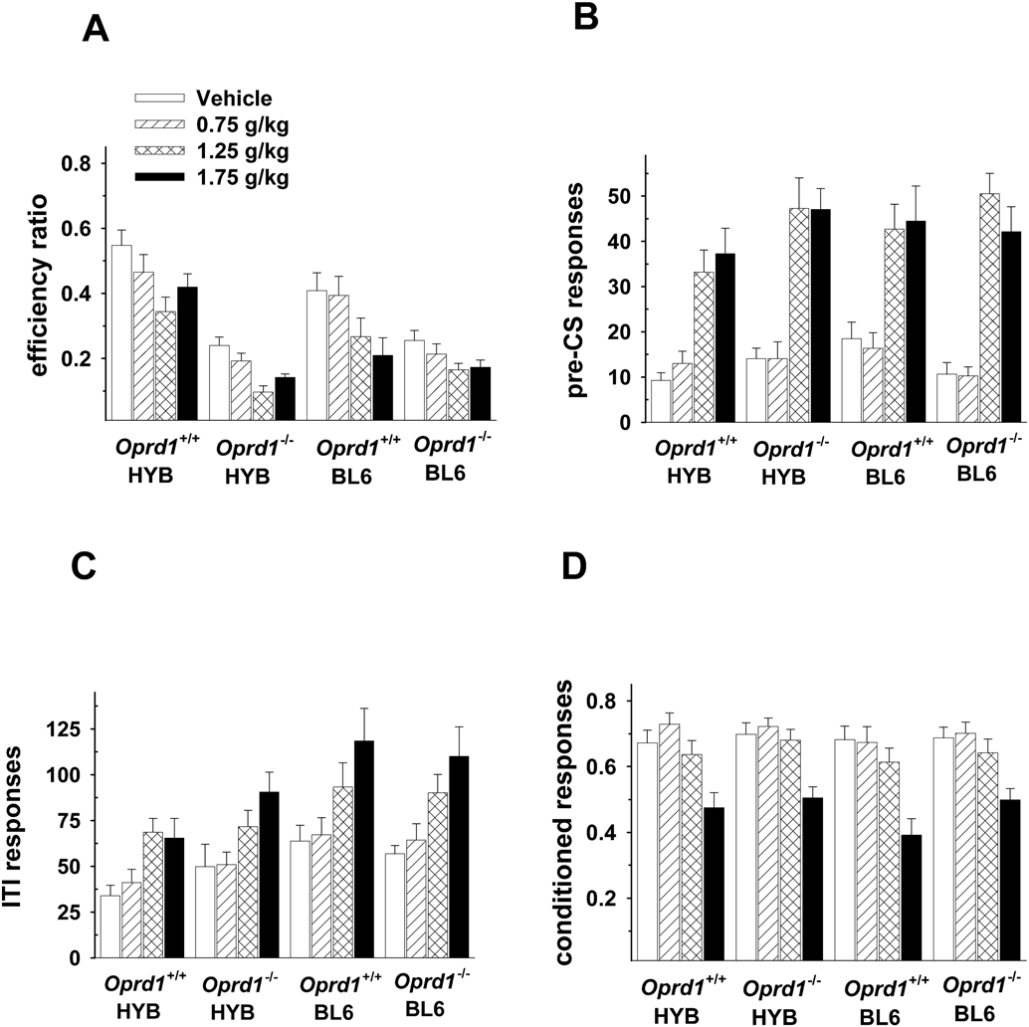
*Oprd1* ^−/−^ and *Oprd1* ^+/+^ mice are impaired by ethanol in the signaled nose poke task. A: Ethanol produced a dose-dependent decrease in efficiency ratios (rewards/nosepokes) in all groups of animals, with knockout mice displaying lower efficiency ratios throughout testing. B: Ethanol increased nose pokes that occurred during the 1–8 s pre-CS period and during the inter-trial interval (ITI) (C). Both wild-type and knockout mice on a BL6 background exhibited higher rates of responding than HYB mice during the ITI but this effect did not interact with dose or with group. D: Ethanol altered conditioned responses in both *Oprd1* ^+/+^ and *Oprd1* ^−/−^ mice, although only the highest dose (1.75 g/kg) produced an effect. *Oprd1^+/+^* HYB, *n = *9*; Oprd1^ +/+^* BL6, *n* = 12; *Oprd1 ^−/−^* HYB, *n = *8*; Oprd1 ^−/−^* BL6, *n = *10.

Ethanol administration had markedly different effects in *Oprd1* ^−/−^ than in *Oprm1* ^−/−^ mice (Fig. 5). Specifically, ethanol decreased efficiency ratios in both wild-type and knockout mice [F(3,222) = 16.11, p<.01], although the increased impulsivity in *Oprd1* ^−/−^ mice was maintained throughout these sessions [F(1,74) = 32.45, p<.01] (Fig. 5A). Ethanol also produced similar effects in wild-type and knockout mice as well as the number of responses that occurred during the pre-CS periods (Fig. 5B) [F(3,222) = 58.71, p<.01], the ITI (Fig. 5C)[F(3,222) = 21.37, p<.01] and as well as on conditioned responses (Fig. 5D) [F(3,222) = 89.95, p<.01]. BL6 animals of both genotypes exhibited higher rates of responding during the ITI period [F(1,74) = 6.76, p<.05] but none of the other main effects or interactions were statistically significant.

## Discussion

This study demonstrates that mice lacking mu-opioid receptors exhibit decreased motor impulsivity whereas those lacking delta-opioid receptors show increased motor impulsivity. Thus, our results reveal an unforeseen role of endogenous opioid receptor activity in disinhibition and suggest that mu opioid receptors promote, whereas delta opioid receptors inhibit, impulsive behaviors. Future studies, using a similar approach, will determine whether kappa receptor activity also influences motor impulsivity.

The increased ability to withhold a motor response in *Oprm1* ^−/−^ mice occurred when reward-related learning remained intact. The latter observation seems to contrast other reports of reward reduction in these animals [9]. These studies, however, examined the effect of mu-opioid receptor gene deletion on the hedonic properties of abused drugs whereas our study tested nosepoking responses to a food reward which is not altered in mu-opioid receptor knockout mice [19]. Importantly, the fact that mu-receptors modulate motor impulsivity independently from conditioned reward in the signaled nose poke tasks is consistent with the notion that reward and impulsivity are mediated by different neural systems [13]. Changes in drug reward and impulsivity, therefore, are separate processes that interact to influence the development of addiction: combined with previous evidence, our findings highlight the fact that mu-opioid receptors play a key role in both processes. These receptors, therefore, not only mediate initial drug reward but may also be implicated in further behavioral changes, such as loss of control, that occur in chronic drug abusers.

Our findings that mice lacking mu-opioid receptors are less impulsive has direct relevance to heroin addiction. At least a small population of heroin addicts exhibit greater binding potential of mu-opioid receptors [20], suggesting that these individuals should display the opposite behavior to that of our knockout mice (i.e., increased impulsivity). The evidence for this hypothesis is mixed: although heroin addicts show clear deficits in cognitive or choice impulsivity [21]–[23], their performance on tests that measure motor impulsivity is less clear. Some of these discrepancies may reflect drug history as pure heroin abusers [24], [25], but not heroin addicts who also abuse other substances [23], exhibit these deficits. Motor deficits in opiate abusers may also depend on current drug use because heroin addicts maintained on methadone show motor impulsivity deficits that are not apparent in abstinent abusers [26]. Although the relationship between the behavior of *Oprm1* ^−/−^ mice and heroin addicts is not straightforward, our results fit well with evidence that opioid antagonists enhance control of motor responses in healthy participants [27] and may be effective treatment in impulse-control disorders [28].

The decreased ability of *Oprd1* ^−/−^ mice to withhold a motor response is intriguing for two reasons. First, these data add to increasing evidence that mu and delta opioids receptors have opposing roles in many behavioral responses. These include anxiety and depressive-like behaviors [12], ethanol self-administration [29] and a conditioned place preference to cannabinoids [10]. The two receptors also differentially regulate mesolimbic DA tone [30]. Second, clinical research and practice have probed the function of mu opioid receptors using morphine-derived compounds for decades. In contrast, delta opioid receptor pharmacology is less well developed and many aspects of delta receptor function remain unexplored. Our findings suggest that, in addition to their anxiolytic and antidepressant effects, delta receptor agonists are of potential interest for impulse control disorders. With regards to drug abuse, delta opioid receptors may minimize changes in emotional state and impulsivity, both of which develop under chronic drug abuse and contribute to relapse.

Consistent with previous evidence [31], [32], strain differences in impulsivity emerged in that BL6 mice were more impulsive than the HYB groups. The fact that basal motor impulsivity was distinct in the two wildtype strains emphasizes the importance of examining knockout mice on both background. Strain conferred a performance advantage in all groups, with the exception of *Oprm1^−/−^* mice, probably because these animals were already responding at optimal levels. Indeed the performance of *Oprm1^−/−^* mice was so efficient that they made, on average, fewer than 5 pre-CS responses during the 10^th^ training session. Compared to other studies using the same task [15], [33], our animals performed exceptionally well with efficiency ratios of wild-type animals ranging from .31 to .5 at the end of training. In addition to strain differences, we used a visual rather than an auditory cue and liquid rather than pellet sucrose reward. Either or all of these factors may have increased the performance of wild-type mice and make it even more surprising that we observed such a dramatic enhancement in mice lacking the *Oprm1* gene.

We also provide compelling evidence that *Oprm* ^−/−^ mice are insensitive to the acute effects of ethanol in a signaled nose poke task. This fits with evidence that mice lacking the *Oprm1* gene show reduced responses to ethanol in other behavioural tests [34] which may be mediated by the reduction in ethanol-stimulated DA release in the ventral striatum in *Oprm1* ^−/−^ mice [35]. We also found that strain differences, which were apparent during impulsivity testing, disappeared under the influence of ethanol. This probably reflects increased experience with the task rather than a drug-induced dampening of strain differences because BL6 and HYB wild-type mice showed the same level of performance following vehicle injections. Thus, although strain may influence impulsivity measures, the effect is not nearly as robust as the *Oprm1* ^−/−^ and *Oprm1* ^+/+^ difference that was apparent in both drugged and drug-free tests.

Higher activity under the influence of ethanol probably accounts for the increased responses during both pre-CS and ITI periods, producing lower efficiency ratios in both wild-type and *Oprd1* ^−/−^ mice. The reduction in conditioned responses could reflect a cognitive deficit that is manifested as an inability to detect and/or respond to the visual stimulus. Even if this is true, mice were not completely disoriented because responses on the non-reinforced side did not change with ethanol administration (mean responses per session <10 for all groups). In light of these dramatic effects of ethanol in wild-type and *Oprd* ^−/−^ mice, the most striking finding is that the drug was completely ineffective in altering the behavior of mice lacking the *Oprm^−/−^* gene.

Mechanisms by which mu- and delta-opioid receptors regulate motor impulsivity remain to be elucidated: there are several potential neural substrates for opioid-controlled disinhibition. The decreased impulsivity we observed in *Oprm1* ^−/−^ mice could be mediated through an interaction with the subthalamic nucleus (STN), dopamine (DA) D2 receptors in the ventral striatum (VS), and/or excitatory projections from the prefrontal cortex (PFC) to the striatum. Lesions of either the PFC [36] or STN [37] increase impulsivity suggesting that deletion of the *Oprm1* gene may increase activity in either structure. With regards to the STN, this hypothesis is plausible as activation of mu-opioid receptors inhibits excitatory inputs to the STN [38]: the absence of mu-opioid receptors would be associated with increased excitation of STN neurons. The possibility that mice lacking mu-opioid receptors exhibit increased activity in PFC-striatal circuits is less convincing because activation of mu-opioid receptors in the PFC inhibits GABA interneurons that synapse on PFC projections [39]. Removal of opioid-induced inhibition of GABA neurons would decrease activity in PFC projections. On the other hand, a small population of mu-opioid receptors is located on PFC projection neurons [40] so deletion of the *Oprm1* gene could increase PFC activity by directly removing this inhibitory input. Finally, decreased availability of DA D2-like receptors in the VS is associated with high levels of trait impulsivity [41] suggesting that knockout mice may exhibit increased signaling in DA D2 pathways. This idea fits with evidence that mice lacking the *Oprm1* gene show increased DA D2 receptor binding in the striatum and increased activity levels when the receptor is stimulated [42]. Much less is known about delta receptor function in these brain areas, or about D2/delta receptor interactions. In addition, the effect of selective opioid receptor ligands in the STN, PFC or VS on impulsive responding has not been tested directly. The deletion of the *Oprm1* and *Oprd1* genes may, together, alter inhibitory mechanisms through one or more of these neural systems.

An important remaining question is whether deletion of the *Oprm1* or *Oprd1* genes affect other measures of impulsivity, such as delay-discounting or reflection impulsivity. The latter is particularly interesting because it represents a cognitive marker for substance dependence that does not recover with prolonged abstinence and is associated with multiple drugs of abuse [43]. The importance of impulsivity in addiction is emphasized further by a relationship between level of abuse and treatment retention [44]. Finally, beyond addiction, the role of the *Oprm1* and *Oprd1* genes in impulsivity has implications for understanding and treating attention deficit hyperactivity disorder, eating disorders, gambling and other disorders of impulse control.
